# Susceptibility and Severity of COVID-19 Are Both Associated With Lower Overall Viral–Peptide Binding Repertoire of HLA Class I Molecules, Especially in Younger People

**DOI:** 10.3389/fimmu.2022.891816

**Published:** 2022-07-07

**Authors:** Hamid Reza Ghasemi Basir, Mohammad Mahdi Majzoobi, Samaneh Ebrahimi, Mina Noroozbeygi, Seyed Hamid Hashemi, Fariba Keramat, Mojgan Mamani, Peyman Eini, Saeed Alizadeh, Ghasem Solgi, Da Di

**Affiliations:** ^1^ Department of Pathology, School of Medicine, Hamadan University of Medical Sciences, Hamadan, Iran; ^2^ Brucellosis Research Centre, Hamadan University of Medical Sciences, Hamadan, Iran; ^3^ Department of Immunology, School of Medicine, Hamadan University of Medical Sciences, Hamadan, Iran; ^4^ Department of Radiology, School of Medicine, Hamadan University of Medical Sciences, Hamadan, Iran; ^5^ Anthropology Unit, Department of Genetics and Evolution, University of Geneva, Geneva, Switzerland

**Keywords:** COVID-19, HLA, overall binding repertoire, SARS-CoV-2-derived peptides, HLA binding prediction

## Abstract

An important number of studies have been conducted on the potential association between human leukocyte antigen (*HLA*) genes and COVID-19 susceptibility and severity since the beginning of the pandemic. However, case–control and peptide-binding prediction methods tended to provide inconsistent conclusions on risk and protective *HLA* alleles, whereas some researchers suggested the importance of considering the overall capacity of an individual’s HLA Class I molecules to present SARS-CoV-2-derived peptides. To close the gap between these approaches, we explored the distributions of *HLA-A*, *-B*, *-C*, and *-DRB1* 1st-field alleles in 142 Iranian patients with COVID-19 and 143 ethnically matched healthy controls, and applied *in silico* predictions of bound viral peptides for each individual’s HLA molecules. Frequency comparison revealed the possible predisposing roles of HLA-A*03, B*35, and DRB1*16 alleles and the protective effect of HLA-A*32, B*58, B*55, and DRB1*14 alleles in the viral infection. None of these results remained significant after multiple testing corrections, except HLA-A*03, and no allele was associated with severity, either. Compared to peptide repertoires of individual HLA molecules that are more likely population-specific, the overall coverage of virus-derived peptides by one’s HLA Class I molecules seemed to be a more prominent factor associated with both COVID-19 susceptibility and severity, which was independent of affinity index and threshold chosen, especially for people under 60 years old. Our results highlight the effect of the binding capacity of different HLA Class I molecules as a whole, and the more essential role of *HLA-A* compared to *HLA-B* and *-C* genes in immune responses against SARS-CoV-2 infection.

## Introduction

The ongoing Coronavirus Disease 2019 (COVID-19) pandemic caused by severe acute respiratory syndrome coronavirus 2 (SARS‐CoV‐2) has caused global public health and economic disasters. By March of 2022, SARS‐CoV‐2 spread in more than 200 countries with over 270 million cases, leading to 5.9 million deaths (1). Currently, researchers are putting all their efforts to better understand this novel viral infectious disease, which varies in terms of geographical distribution, mortality, and severity of symptoms around the world (2). Older age, male sex, and comorbidities were identified as important risk factors of COVID-19 pathogenesis (3), and its outcome may also be shaped by both the genetic landscape of an individual and the population (4, 5).

The human leukocyte antigen (*HLA*) gene complex on the short arm of chromosome 6 contains the most polymorphic gene cluster of the human genome and plays a substantial role in induction of immune responses against pathogens. In the case of viral infections, classical HLA Class I (HLA-A, -B, and -C) molecules on the surfaces of infected cells present virus-derived peptides to CD8^+^ cytotoxic T lymphocytes, leading to their elimination by the latter, whereas classical HLA Class II (HLA-DR, HLA-DP, and HLA-DQ) molecules display such degradation products for stimulation of CD4^+^ helper T lymphocytes, generating production of neutralizing antibodies and inflammatory cytokines (6). Conformational variation of HLA molecules, especially in the peptide-binding cleft, affects more or less the binding repertoire of virus-derived peptides. It is thus unsurprising that a great number of *HLA* alleles and SNPs have been associated with viral infections (7, 8).

In this context, a large body of studies have already been accomplished on the potential association of *HLA* genes with COVID-19 since the outbreak of this pandemic (9). Based on their *in silico* predictions of HLA binding affinity to SARS-CoV-2 peptides, Nguyen et al. (10) compared the numbers of predicted bound peptides among HLA Class I molecules and argued that HLA-B*46:01 might be the most susceptible allele for SARS-CoV-2 infection, whereas B*15:03 might be the most protective one. However, among the dozens of case–control studies investigating potential HLA–COVID-19 association, few results have explicitly supported the prediction data. Through frequency comparisons between patients and controls, multiple *HLA* Class I and Class II alleles were reported as risk or protective factors in these studies, strongly depending on populations, and most results became insignificant after multiple testing corrections (11–16). In contrast, no significant association between *HLA* alleles and the disease was observed in studies focusing on South Asia, Brazil, Italy, Spain, and Germany (17–20). Likewise, using a large Ashkenazi sample, Ben Shachar et al. (21) did not find any significant association among 66 most common *HLA-A*, *-B*, *-C*, *-DQB1*, and *-DRB1* alleles, and concluded that if any *HLA* association exists with the disease, it would be very weak.

At the population level, models on *HLA* evolution suggested that *HLA* homozygosity might be associated with poorer disease resistance (22). Thereby, de Marco et al. (17) noticed that homozygosity at *HLA-A* locus was associated with COVID-19 susceptibility but not severity, and Iturrieta-Zuazo et al. (23) observed a higher proportion of *HLA-A* and *-C* homozygotes in patients with severe COVID-19 than in those with the moderate form of the disease. For them, what matters might not be any particular *HLA* polymorphism, but rather the overall capacity of HLA molecules to bind SARS-CoV-2-derived peptides. Among *HLA* genes, La Porta and Zappeti (24) identified two sets of haplotypes with strong and weak predicted binding capacity. Shkurnikov et al. (15) further developed a principal component-based risk score to measure the aggregate capacity of HLA Class I molecules to present SARS-CoV-2 peptides for each individual, and showed a significantly higher score in the group of relatively younger deceased COVID-19 patients (≤60 years old) compared to elderly ones (>60 years old). However, a direct link between one’s overall repertoire of different HLA molecules and COVID-19 susceptibility and severity was yet to be established. It was also necessary to close the gap between case–control results and prediction data, as did Arora et al. (25) for HIV-1. Here, we explored the distributions of *HLA* Class I and Class II alleles and haplotypes in 285 Iranian patients with COVID-19 and healthy controls. In addition, to direct comparisons of allele and haplotype frequencies between patients and controls and between patient subgroups defined by disease severity, we paid more attention to the genetic diversity, and studied the overall viral peptide repertoire of individuals using predicted binding-affinity data.

## Materials and Methods

### Case–Control Study Subjects

This retrospective cohort study was conducted with the approval of the institutional Ethics Committee, Hamadan University of Medical Sciences (IR.UMSHA.REC.1399.005). Blood samples were collected from 142 COVID-19 patients who were admitted to Sina University Hospital of Hamadan in the northwestern part of Iran between July and August 2020. As controls, 143 ethnically matched healthy volunteers were recruited among blood donors during the same period and from the same geographic area. Among them, 135 were negative for IgG antibody (Quanti SARS-CoV-2 Anti-spike IgG antibody, Pishtazteb Co. Tehran, Iran) and without any symptoms related to COVID-19, and the 8 others not being tested did not have any symptoms since the beginning of the pandemic.

Diagnosis and confirmation of SARS-CoV-2 infection were carried out based on the presence of viral RNA in the nasopharyngeal swab samples (laboratory confirmed disease) and/or observation of the radiological changes in CT scan as well as known clinical presentations of COVID-19 for the suspected cases. In terms of disease severity, the patients were classified into three subgroups, namely, 46 hospitalized cases with the moderate form of the disease but not requiring admission at the intensive care unit (ICU) and not requiring supplemental oxygen (“mild/moderate” subgroup), 52 patients with severe COVID-19 who require supplemental oxygen at the ICU (“severe” subgroup), and 44 patients with critical COVID-19 at the ICU that required invasive mechanical ventilation (“critical” subgroup). Classification of the patients was based on the local guidelines and radiological findings as follows: The radiologist evaluated all five lobes of both lungs for the presence of inflammatory abnormalities including ground-glass opacities, mixed ground-glass opacities, and consolidation, according to the method presented by Li et al. (26) and Li et al. (27). In terms of the percentage of involvement, a score of 0.0 to 4.0 was considered for each lobe: 0 (0%), 1 (1%–25%), 2 (26%–50%), 3 (51%–75%), or 4 (76%–100%). Then, the total severity score (TSS) was calculated by summing the points of the five lobes, which ranges from 0 to 20. A TSS equal to or less than 3 was considered as *mild* involvement; 4 to 7, *moderate*; and ≥ 8, *severe* (28). Moreover, in the presence of imaging criteria of acute respiratory distress syndrome (ARDS) including ground-glass attenuation associated with traction bronchiolectasis or bronchiectasis, airspace consolidation associated with traction bronchiolectasis or bronchiectasis, crazy-paving pattern, and honeycombing, or in the presence of complications such as pneumothorax, a TSS equal to or more than 8 was considered as *critical* disease (27). Due to their similarities, the severe and critical subgroups were also combined as Severe/Critical for some analyses.

Age and sex information were recorded for both controls and patients. The main clinical characteristics and information of comorbidities (e.g., diabetes, renal disease, liver disease, hypertension, cardiovascular disease, malignancies, and other infectious diseases) were documented using medical records for all patients (**Supplementary Table 1**).

### *HLA* Genotyping

Primarily, genomic DNA was extracted from EDTA containing peripheral blood samples by implementing an improved salting-out method. In the next step, genotypes of *HLA-A*, *-B*, *-C*, and *-DRB1* loci for all COVID-19 patients and *HLA-A*, *-B*, and *-DRB1* loci for healthy controls were determined by polymerase chain reaction with a sequence-specific primer (PCR-SSP) method using low-resolution *HLA-A-B-C* and *HLA-A-B-DR* SSP kits (Olerup SSP^®^A-B-C and Olerup SSP^®^A-B-DR SSP Combi Trays, Stockholm, Sweden) according to the manufacturer’s protocols. Unfortunately, HLA-C locus was not typed for controls. Specific *HLA-A*, *-B*, *-C*, and *-DRB1* allele families (1st-field, which will be referred to as “alleles” for reason of simplicity) were determined by SCORE software, v5.00.80.02 T/07 provided by the company (29). The *HLA-DRB1* data of patients have already been reported recently (12) in comparison with a different set of controls.

### Binding Affinity Predictions

To study the difference in binding repertoires of viral peptides between HLA molecules, we extracted the whole proteome of a B.4 SARS-CoV-2 variant (30) submitted to GenBank (MT994849.1). This variant was identified to be the major viral cluster during March–July 2020 in the area (31). A total of 9,660 different 9-mer and 9,594 different 15-mer peptides were obtained, the two lengths representing the most common binders of HLA Class I and Class II molecules, respectively. According to a list of 2nd-field *HLA* alleles we prepared, the binding affinity of each corresponding Class I molecule (HLA-A, -B, and -C) to each 9-mer peptide and that of each Class II molecule (HLA-DR) to each 15-mer peptide were predicted by applying the state-of-the-art prediction tools netMHCpan 4.1 (32) and netMHCIIpan 4.0 (32, 33), respectively.

The list of our 2nd-field *HLA* alleles includes all those considered as “commonly” distributed in worldwide population (observed in at least five populations) that we defined in a previous study (34). In order to adapt the prediction results for 2nd-field alleles to our 1st-field data of patients and controls, we assigned each observed 1st-field allele to the most probable 2nd-field one, using as a reference a set of 2nd-field *HLA-A*, *-B*, and *-DRB1* allele frequency data recently reported for 90 Iranians from Yazd province in the center of Iran (35), who share similar ethno-linguistic background with Hamadan people (**Supplementary Table 2**). To avoid sampling bias, we also performed a random assignment procedure, during which each observed 1st-field allele was assigned to a random common 2nd-field allele sharing the same 1st-field number.

Data of two indices, i.e., IC_50_ and %Rank, measuring binding affinity were retrieved from the raw output of netMHCpan and netMHCIIpan, respectively. Both indices may be used to determine if a specific peptide can be considered as a binder to an HLA molecule. Based on the thresholds suggested for IC_50_ and %Rank to define weak (IC_50_: 500 nm; %Rank: 2 for Class I molecules, 10 for Class II molecules) and strong binders (IC_50_: 50 nm; %Rank: 0.5 for Class I molecules, 2 for Class II molecules), we computed for each HLA molecule the numbers of predicted weak and strong bound peptides derived from SARS-CoV-2 proteome. We kept the results from both indices and both thresholds to control possible bias introduced by the choice of these thresholds, which may actually vary among HLA molecules (36, 37). In reality, IC_50_ has been used in most of the aforementioned prediction studies focusing on *HLA*–COVID-19 associations, whereas %Rank seems to be more realistic according to recent arguments (38, 39). Number of distinct weak and strong binders predicted for each HLA molecule was then computed according to each of the two indices. To measure the overall HLA capacity to bind SARS-CoV-2-derived peptides, we further computed, for each patient and control, the cumulative numbers of weak and strong binders, namely, the overall viral peptide repertoire sizes of her/his HLA molecules encoded by (1) both *HLA-A* alleles (n_A_), (2) both *HLA-B* alleles (n_B_), (3) all four *HLA-A* and *-B* alleles (n_AB_), and (4) both *HLA-DR* (n_DR_) alleles. For each patient, since the *HLA-C* genotype was available, those numbers for (5) both *HLA-C* alleles (n_C_) and (6) all six *HLA-A*, *-B*, and *-C* (n_ABC_) alleles were equally included.

### Statistical Analyses

By the use of the GENE[RATE] tools available on HLA-net (40), we first tested Hardy–Weinberg equilibrium in the control group for each locus, then estimated allele frequencies as well as two-locus haplotype frequencies. We further computed, for each locus, two slightly different measures of genetic diversity (41), i.e., heterozygosity index (h) and frequency of homozygotes.

A general comparison of *HLA* allele distribution between the patient and control groups was performed by computing pairwise F statistics (F_ST_) using Arlequin (42) v3.5.2.2, the significance of which is accessed by a procedure of 10,000 permutations. Considered as two samples, patients and controls were also compared to the Yazd Iranian sample (43). Comparisons of the frequencies of specific *HLA* genotypes, alleles, and haplotypes between patient and control groups and among patient subgroups were performed using Fisher’s exact test (44), and their corresponding odds ratio (OR) was estimated with 95% confidence interval. *p*-value less than 0.05 was considered as statistically significant, and the Benjamini–Hochberg method for multiple comparisons was used to control the false discovery rate (45).

Distributions of the numbers of predicted bound peptides (n_A_, n_B_, n_AB_, n_DR_, n_C_, and n_ABC_) were summarized by using kernel density estimation (46), and their difference was compared by using two-tailed Wilcoxon test (47). The relationship between severity and the overall repertoires of HLA molecules was further studied through generalized linear models (48).

To study the influence of age, the widely observed risk factor of COVID-19, comparisons between patients and controls and between patient subgroups were also performed for individuals under 60 years old.

All analyses were performed with R (49) v4.1.2 implemented in RStudio (50) unless otherwise specified. Data visualization was accomplished by using the ggplot2 package (51) v3.3.5.

## Results

### Age, Sex, and Comorbidities

Significant difference was found for age (*p* < 0.0001) between patients and controls, which was not the case for sex (*p* > 0.05), though more male patients were found among patients (77/142 vs. 68/143). Since the healthy controls had no comorbidities, comparison of the main clinical features and demographic factors was only performed between the three patient subgroups (**Supplementary Table 1**). Significant differences were also observed for age (*p* < 0.001) and most clinical indices and comorbidities, but not for sex, either. It is interesting to note that there is a much higher proportion of patients with olfactory dysfunction (OD) as well as a decreased proportion of patients with negative PCR results in the critical subgroup, the former being in contrast with previous reports that show that OD appeared to be more prevalent in patients with mild-to-moderate symptoms (52).

### Comparison of *HLA* Diversity and Allele Distributions Between Patients and Controls and Among Patient Subgroups

**Figure 1:**
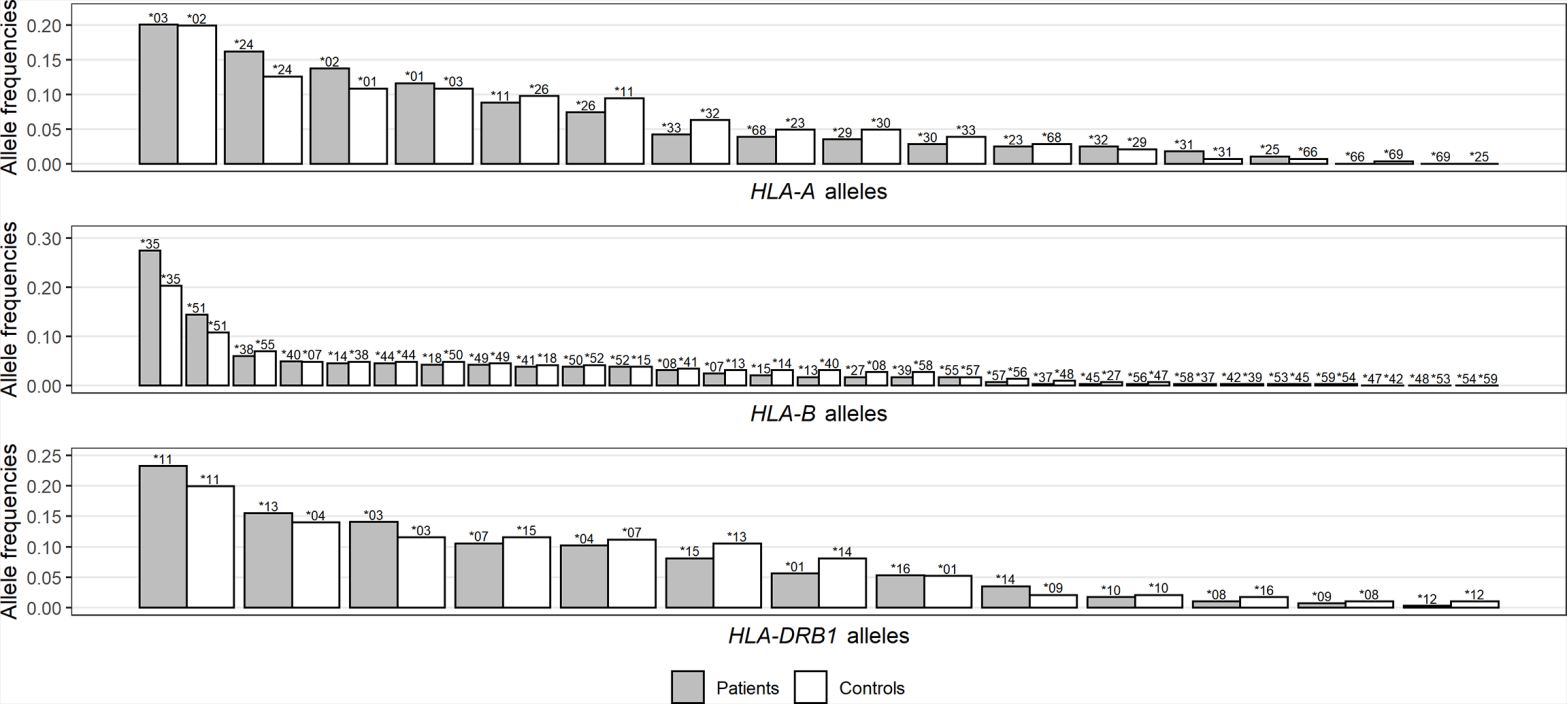
Distribution comparison of allele frequencies for *HLA-A* **(A)**, *-B* **(B)**, and *-DRB1* **(C)** loci between patients and controls. Noting that *HLA* alleles for each group are ranked by frequencies and represented by bars of different lengths with their names on top, each bar pair does not necessarily correspond to the same allele pair.

At the within-population level, no deviation from Hardy–Weinberg equilibrium was observed in the control group for any locus we analyzed (**Supplementary Table 3**). At a first glance, the patient group had lower heterozygosity (h) for all three *HLA* loci (**Table 1**), and the differences became more visible when displaying the distribution of *HLA* frequencies in the two groups (**Figure 1**). With the observed alleles ranked by frequency for each locus, patients had more alleles with high frequencies and less alleles with intermediate frequencies than controls, implying an excess of homozygosity. Accordingly, a higher proportion of homozygotes was found among patients than controls for *HLA-A* and *-B*, which was not the case, however, for *HLA-DRB1* (**Table 1**), and none of these differences in numbers was significant according to Fisher’s exact test. Interestingly, when looking at genotypes of specific alleles, we noticed that the homozygotes of some specific alleles were unevenly distributed in patients and controls, with an obvious concentration of A*03, A*24, and B*35 homozygotes in the patient group (**Supplementary Figure 1**).

**Table 1:** Heterozygosity and proportion of homozygotes at each HLA locus computed for each group and subgroup.

Loci	Indices	All patients	All controls	Mild/Moderate	Severe	Critical	Severe/Critical
**HLA-A**	h	0.8810	0.8909	0.8693	0.8889	0.8670	0.8814
	%homozygotes	16.20 (23/142)	11.19 (16/143)	13.04 (6/46)	21.15 (11/52)	13.64 (6/44)	17.71 (17/96)
**HLA-B**	h	0.8820	0.9191	0.8762	0.8852	0.8696	0.8820
	%homozygotes	11.27 (16/142)	5.59 (8/143)	6.52 (3/46)	17.31 (9/52)	9.09 (4/44)	13.54 (13/96)
**HLA-C**	h	0.8700	–	0.8715	0.8711	0.8489	0.8665
	%homozygotes	17.61 (25/142)	–	13.04 (6/46)	19.23 (10/52)	20.45 (9/44)	19.79 (19/96)
**HLA-DRB1**	h	0.8663	0.8799	0.8542	0.8707	0.8502	0.8662
	%homozygotes	11.27 (16/142)	16.78 (24/143)	8.70 (4/46)	13.46 (7/52)	11.36 (5/44)	12.50 (12/96)

Among patient subgroups, heterozygosity did not show any consistent difference. In contrast, proportions of *HLA-A*, *-B*, *-C*, and *-DRB1* homozygotes all increased with severity, especially when severe and critical subgroups were combined (**Table 1**). Homozygotes of specific alleles were not compared among subgroups because of low counting numbers.

At the among-population level, when we looked at the pairwise F_ST_ between patients, controls, and Yazd Iranians (**Supplementary Table 4**), the highest values were found between patients and Yazd Iranians for the three loci, and all with significant *p*-values. The controls were genetically closer to Yazd Iranians, much clearer for *HLA-A*, where the F_ST_ was not significant, but less obvious for *HLA-DRB1*.

### Comparison of *HLA* Allele and Haplotype Frequencies Between Patient and Control Groups and Among Patient Subgroups

**Figure 2:**
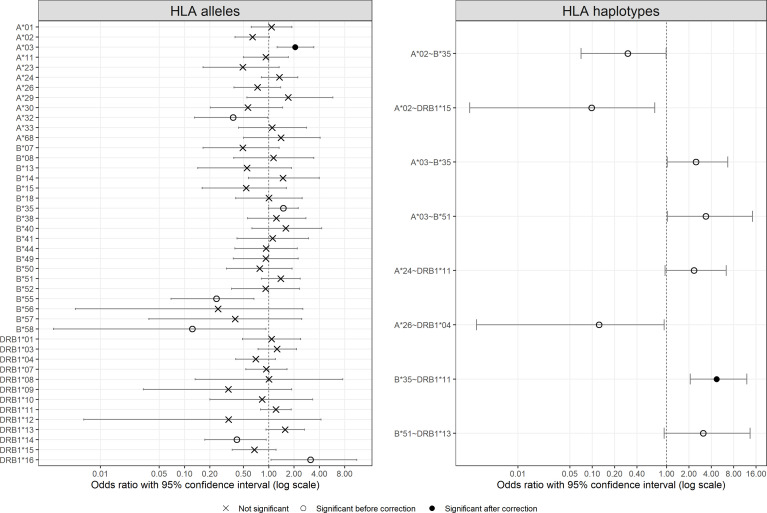
Odds ratio with 95% confident intervals for *HLA* alleles and *HLA* two-locus haplotypes. For haplotypes, only those with significant results are shown. Significance is represented by different symbols: crosses for non-significant results; circles for significant results before correction and dots for significant results after correction.

**Figure 2** visualizes odds ratios with 95% confidence interval estimated for *HLA-A*, *-B*, and *-DRB1* alleles and results of Fisher’s exact test between patients and controls (detailed results are listed in **Supplementary Table 5**). Among *HLA* alleles, A*03 (OR = 2.06, *p* = 0.0025), B*35 (OR = 1.49, *p* = 0.0494), and DRB1*16 (OR = 3.13, *p* = 0.0237) were significantly more frequent in the patient group (susceptible), whereas A*32 (OR = 0.38, *p* = 0.0388), B*55 (OR = 0.24, *p* = 0.0033), B*58 (OR = 0.12, *p* = 0.0376), and DRB1*14 (OR = 0.42, *p* = 0.0300) were significantly more prevalent in the control group (protective). After multiple testing corrections, only the result for A*03 (*p*_C_ = 0.0403) remained significant. Concerning severity, A*03, A*32, B*27, B*39, B*55, and DRB1*16 showed differences among the control group and patient subgroups, the frequencies of which did not, however, seem to be associated with severity (**Supplementary Figure 2**).

In the same way, **Figure 2** also shows the odds ratios with 95% confidence interval for *HLA-A*~*B*, *B*~*DRB1*, and *A*~*DRB1* haplotypes more frequent than 1% in at least one group (detailed results are listed in **Supplementary Table 6**, and a full list of estimated two-locus haplotype frequencies is available in **Supplementary Table 7**). The majority of the haplotypes with significant results were composed of one or two of the aforementioned alleles with different frequencies in the two groups, such as A*02~B*35, A*03~B*35, and B*35~DRB1*11. Only the result for B*35~DRB1*11 (*p*_C_ = 0.0010) remained significant after multiple testing corrections. Concerning patient subgroups, no haplotype showed an interesting association between frequency and severity (results not shown).

### Comparison of Overall HLA Binding Repertoire Between Patient and Control Groups and Among Patient Subgroups

When *HLA* alleles were ranked according to the numbers of predicted bound peptides derived from SARS-CoV-2 proteome, none of the corresponding alleles detected by frequency comparisons showed any particularity, without extremely low or high values, respectively (**Supplementary Figure 3**; values for each HLA molecule are available in **Supplementary Table 8**). For example, the only allele with significant result after corrections, namely, HLA-A*03, was medium ranked in IC_50_-based lists and low ranked in %Rank-based lists. Actually, the ranking lists depended strongly on indices and thresholds chosen and differed considerably between the results from %Rank and IC_50_.

**Figure 3:**
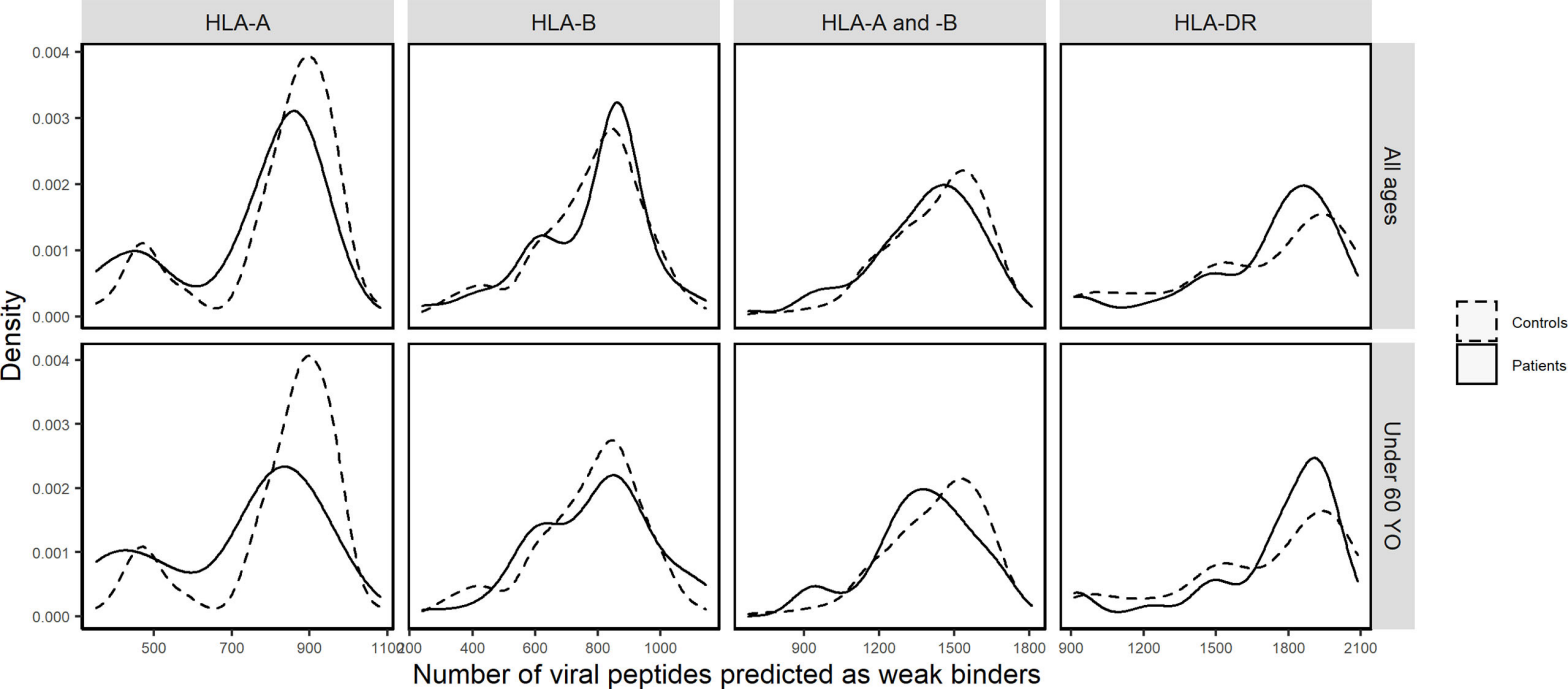
Density distributions of the numbers of SARS-CoV-2-derived peptides predicted by %Rank as weak binders to HLA-A (n_A_), HLA-B (n_B_), HLA-A and -B (n_AB_), and HLA-DR (n_DR_) molecules in patients (solid curves) and controls (dashed curves) of all ages and in those under 60 years old, respectively.

In contrast, by comparing cumulative numbers of different SARS-CoV-2-derived bound peptides at the individual level, patients and controls did show remarkable differences, which were more consistent among indices and thresholds chosen. **Figure 3** visualizes density distributions of these numbers for each of the two groups, taking %Rank index and threshold for weak binders as example, and density charts with different indices and/or thresholds are found in **Supplementary Figures 4**–**6**. Indeed, a higher proportion of patients carried HLA-A molecules predicted to bind only 500 to 750 different viral peptides, whereas more controls carried HLA-A molecules predicted to bind more than 750 different viral peptides. When considered as a risk factor, n_A_ less than 750 had an odds ratio of 2.04 (95% CI: 1.20 to 3.50; Fisher’s exact test *p* = 0.0084). In contrast, the HLA-B molecules showed less difference between the two groups, and the controls only showed a very slightly higher proportion of values around 700. Considering HLA-A and HLA-B together (n_AB_), the patients also show less bound peptides (peak at approximately 1,400) compared to controls (peak at approximately 1,600). As for HLA-DR molecules, the distribution for patients seemed more concentrated compared to controls. Wilcoxon test confirmed significant difference for n_A_ (*p* = 0.0002) but not for n_B_, n_AB_, and n_DR_ (**Table 2**). More interestingly, when only younger (under 60 years old) individuals were included in the comparisons did numbers of predicted bound peptides differ more apparently between patients and controls for n_A_ and n_AB_ (**Figure 3** and **Supplementary Table 4**). In this case, the odds ratio for n_A_ less than 750 increased to 3.01 (95% CI: 1.54 to 5.93; Fisher’s exact test *p* = 0.0014).

**Table 2:** p-values (p < 0.05 in bold) from the two two-tailed Wilcoxon test between patients and controls of all ages or those under 60 years old on numbers of SARS-CoV-2-derived peptides predicted as weak binders of HLA molecules (HLA-A: n_A_; HLA-B: n_B_; HLA-A and HLA-B: n_AB_; HLA-DR: n_DR_) according to %Rank.

	n_A_	n_B_	n_AB_	n_DR_
**All ages**	**0.0002**	0.4836	0.0929	0.7664
**Under 60**	**0.0002**	0.5178	0.0859	0.8726

**Figure 4:**
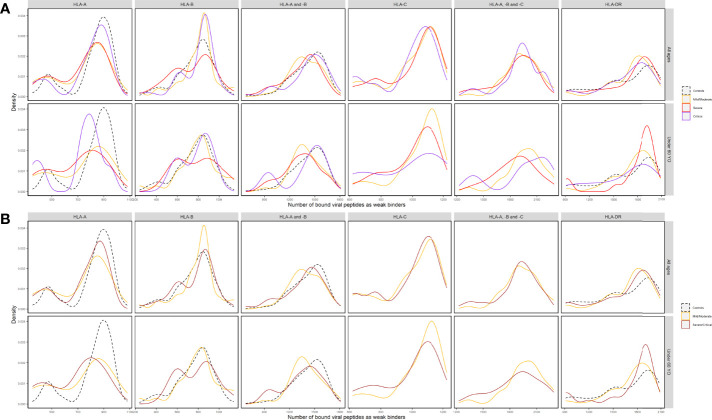
Density distributions of the numbers of SARS-CoV-2-derived peptides predicted by %Rank as as weak binders to HLA-A (n_A_), HLA-B (n_B_), HLA-A and -B (n_AB_), HLA-C (n_C_), HLA-A, -B and -C (n_ABC_), and HLA-DR (n_DR_) molecules **(A)** in three subgroups of patients (Mild/Moderate: solid curves in yellow; Severe: solid curves in red; Critical: solid curves in purple) and controls (dashed curves in black) and **(B)** in two subgroups of patients (Mild/Moderate: solid curves in yellow; Severe/Critical: solid curves in rose) and controls (dashed curves in black) of all ages and in those under 60 years old, respectively. Note that n_C_ and n_ABC_ data were not available for controls.

Among the control group and patient subgroups, a smaller HLA-A overall repertoire also seemed to differ, especially when severe and critical subgroups were combined (**Figure 4** for %Rank-based weak binders). Generalized linear models revealed a significant association between n_A_ and severity (*p* = 0.0337, **Table 3**). This is also compatible with IC_50_-based weak and strong binders (*p* = 0.264 and *p* = 0.311, respectively), and with %Rank-based strong binders for which the result was marginally significant (*p* = 0.053; see **Supplementary Table 10**). Visible but not always significant differences were also observed for n_AB_, n_C_, and n_ABC_, and for individuals under 60 years old (**Table 3** and **Supplementary Table 10**).

**Table 3:** p-values (*p* < 0.05 in bold) from generalized linear models (GLM) between severity (control + 3 patient subgroups or control + 2 subgroups) and numbers of SARS-CoV-2-derived peptides predicted as weak binders of HLA molecules (HLA-A: n_A_; HLA-B: n_B_; HLA-A and -B: n_AB_; HLA -C: n_C_; HLA-A, -B and -C: n_ABC_; HLA-DR: n_DR_) according to %Rank for individuals of all ages or those under 60 years old.

		n_A_	n_B_	n_AB_	n_C_	n_ABC_	n_DR_
**Control+3 subgroups**	All ages	**0.0337**	0.8188	0.1947	0.6295	0.6599	0.7310
	Under 60	**0.0010**	0.5749	0.0977	0.1671	0.3515	0.5630
**Control+2 subgroups**	All ages	**0.0084**	0.9310	0.0666	0.8992	0.2496	0.5729
	Under 60	**0.0005**	0.5245	0.0712	0.2001	0.1537	0.3963

Despite minor differences, the repetition of these analyses after the random assignment procedure we designed did not change the results of comparisons (results not shown).

## Discussion

In the present study, we investigated potential associations between *HLA* Class I and Class II genes and susceptibility and/or severity based on a sample of Iranian patients with COVID-19. At a first glance to the sample, older age and comorbidities were both confirmed as major risk factors associated with COVID-19 susceptibility and/or severity (**Supplementary Table 1**), as reported in previous studies.

Direct comparison of allele frequencies between patients and controls revealed possible predisposing roles in SARS-CoV-2 infection for HLA-A*03, B*35, and DRB1*16 alleles and a possible protective effect of HLA-A*32, B*55, B*58, and DRB1*14 alleles. However, among these alleles, HLA-A*03 was the only allele with a significant result after multiple testing correction (**Figure 2**), and none of them seemed to be significantly associated with COVID-19 severity (**Supplementary Figure 2**). Looking into the literature, HLA-A*03 was characterized as a risk factor, along with other alleles that do not correspond to those found in this study, of COVID-19 severity in Spanish (13), Arabic (19), and Iranian patients (53). In contrast, Shkurnikov et al. (15) depicted that A*03:01 decreased the risk of COVID-19. For HLA-DRB1*04, it was suggested to be significantly associated with either susceptibility (19) or severity (54) in different population samples, besides our previous report on *HLA-DRB1* with the same patients but totally different controls (12). Such inconsistency among studies has been attributed to various factors including sampling bias due to small sample sizes, inaccuracy of prediction algorithms, and different genetic background of populations (12). Already noted in previous studies, it also led to the conclusion by some authors that *HLA* might play a small role in COVID-19 susceptibility (20, 21).

From a more general perspective, both F_ST_ and heterozygosity index (h) indicated different distribution patterns of *HLA-A*, *-B*, and *-DRB1* alleles in patients and controls. Patients were significantly differentiated from controls for all loci, whereas the controls were much more similar to Yazd reference population (**Supplementary Table 4**). Compared to controls, patients showed lower genetic diversity at all three loci (**Table 1**), noticeable through their *HLA* allele frequency distribution with an excess of alleles with intermediate frequencies (**Figure 1**), which was more or less reflected by higher proportions of homozygotes for both *HLA-A* and *-B* (**Table 1** and **Supplementary Figure 1**). These results are also compatible with previous findings by Iturrieta-Zuazo et al. (23) and de Marco et al. (17) suggesting lower *HLA-A* diversity in patients. As for severity, homozygote proportions also differed patient subgroups (**Table 1**).

In view of predicted HLA binding affinity, our list of HLA molecules ranked by repertoire of SARS-CoV-2-derived bound peptides (**Supplementary Figure 3**) is in agreement with previous studies (10, 55–57) only when using IC_50_, whereas the ranking list based on %Rank is considerably different. Moreover, the HLA molecules predicted to bind the most and the least peptides do not correspond to the ones detected by frequency comparisons (**Figure 2** and **Supplementary Figure 1**). On the other hand, by computing the aggregate number of different bound viral peptides by one’s HLA molecules, namely, the overall viral peptide repertoire, a significantly higher proportion of patients’ HLA-A molecules were predicted to present less SARS-CoV-2-derived peptides (n_A_) compared to controls. A similar tendency was also visible but less significant for HLA-A and -B overall repertoire (n_AB_), but not for HLA-B overall repertoire (n_B_; **Table 2**, **Supplementary Table 9**, **Figure 3** and **Supplementary Figures 4**–**6**). When considered as a risk factor, n_A_ less than 750 (%Rank-based weak binders) had an odds ratio of 2.04, higher than that computed for most specific *HLA* alleles. Furthermore, n_A_ was associated with disease severity (**Figure 4A**), confirmed by significant correlation from linear modelling (*p* < 0.05), especially when severe and critical subgroups were combined (**Figure 4B**). Though not significant, HLA-A and -B (n_AB_), HLA-C (n_C_), and HLA-A, -B, and -C (n_ABC_) also showed slightly lower numbers of binders in patients (**Figures 3**, **4**). These results support, independently from indices and thresholds, the hypothesis that the overall peptide repertoire of HLA Class I molecules may be a more influencing factor compared to any specific *HLA* allele on both SARS-CoV-2 infection and disease development, and suggest a more prominent role of *HLA-A* compared to *HLA-B* and *-C*.

In contrast, HLA-DRB1 overall repertoire did not seem to be associated with either COVID-19 susceptibility or severity (**Figures 3**, **4**), which is consistent with previous studies (15, 54) and compatible with the fact that HLA Class II molecules are less directly involved with initial reactions against viral infections.

More interestingly, when only younger (<60 years old) individuals were included in the comparisons, the overall repertoires differed more visibly between patients and controls and patient subgroups for n_A_ and n_AB_, though the difference was not always more significant, probably due to reduced individual numbers (**Figures 3**, **4**, **Supplementary Figures 4**–**9**, **Tables 2**, **3**, **Supplementary Tables 9, 10**). The odds ratio for n_A_ less than 750 increased to 3.01 (*p* = 0.0014), indicating that for younger people, smaller overall repertoire might be a more important risk factor. Actually, age itself is an essential factor on the HLA peptide binding capacity since *HLA* expression was reported to be negatively associated with age (58). Among elderly people, larger binding repertoires would barely compensate for their decreased absolute numbers of HLA molecules expressed on cell surface. In addition, most elderly people in patients suffered from one or several comorbidities (**Supplementary Table 1**). These might be among the main reasons why the overall repertoire of one’s HLA-A molecules became a more prominent factor among younger people.

It has long been documented that the current *HLA* variation has been the result of long-term pathogen-mediated balancing selection (59–61). Consequently, molecular and functional divergency is both remarkable among *HLA* alleles observed in modern human populations (34, 62), making the system surprisingly resistant to potential loss of gene diversity (63). The smaller overall viral peptide repertoire predicted for HLA-A and other Class I molecules in a higher proportion of patients would rather be due to a concentration of specific alleles (**Supplementary Figure 1**) with lower binding capacity than the slightly lower genetic diversity or homozygote proportions (17, 23).

To sum up, we suggest that a smaller overall viral peptide repertoire would be a more general risk factor to viral infection, whereas the risk or protective effect of specific HLA molecule(s) might be both population- and pathogen-specific. In a population, each individual may be inevitably more vulnerable to certain specific pathogens due to the lower overall binding capacity for one or several *HLA* genes, but more resistant to others, making the population as a whole more resistant against any diseases, as demonstrated by the results of Barquera et al. (55). As a result, at the population level, it would be more difficult to detect evolutionary signatures on particular *HLA* alleles associated with either susceptibility or resistance to diseases, due to selection from multiple pathogens simultaneously (64, 65).

Unfortunately, in the current study, higher-resolution-level *HLA* data were not available, and *HLA-C* was not typed for controls. In this context, we designed a procedure of assignment to adapt the 1st-field genotype data to 2nd-field prediction results, using high-resolution *HLA* data from a Yazd Iranian population as reference. As for *HLA-C*, the gene was previously suggested to be much less expressed and display less unique peptide repertoire compared to *HLA-A* and *-B* (34, 63), and the latter was again confirmed by the overall number of binders to HLA-A, -B, and -C molecules (n_ABC_; **Figures 4**, **Supplementary Figures 7** **–** **9**). Nevertheless, synthetic analyses including high-resolution *HLA* Class I case–control data from different populations will be necessary. It will also be interesting to consider virus immunogenic epitopes and the expression level of different viral proteins to better estimate the overall binding capacity of HLA molecules to SARS-CoV-2-derived peptides.

In conclusion, despite the fact that several specific *HLA* Class I and Class II alleles/haplotypes, notably HLA-A*03, have been identified to be associated with COVID-19 infection in the Iranian cohort we studied, the overall repertoire of one’s HLA-A molecules and, to a lesser extent, that of one’s HLA Class I molecules to present SARS-CoV-2-derived peptides seem to be a more prominent factor in both susceptibility and severity of the disease, especially for younger people. Inconsistent reports from different studies would have been more related to population-specific combination patterns of *HLA* alleles than to variations in patients’ clinical features and experimental approaches. These findings would also be enlightening to review, from a functional aspect, previously reported associations between *HLA* alleles and other pathogens, particularly human viruses.

## Data Availability Statement

The datasets presented in this study can be found in online repositories. The names of the repository/repositories and accession number(s) can be found in the article/**Supplementary Material**.

## Ethics Statement

This retrospective cohort study was conducted with the approval of the institutional Ethics Committee, Hamadan University of medical sciences (IR.UMSHA.REC.1399.005). The patients/participants provided their written informed consent to participate in this study.

## Author Contributions

HB, GS, and MMM launched the project. GS, SE, MN, and DD designed the study. MMM, SH, FK, MM, and PE collected and provided the samples. SE and MN performed laboratory testing and HLA typing work. GS performed case–control comparisons and DD completed binding predictions and other analyses. DD, GS, and HB wrote the manuscript. HB acquired the funding. All authors contributed to the article and approved the submitted version.

## Funding

This work was supported financially by Vice-Chancellor for Research and Technology, Hamadan University of Medical Sciences (Grant No: 14000207868), Hamadan, Iran, and the Swiss National Science Foundation (Grant Nos. #31003A_144180 and #310030_188820 to Alicia Sanchez-Mazas), Switzerland.

## Conflict of Interest

The authors declare that the research was conducted in the absence of any commercial or financial relationships that could be construed as a potential conflict of interest.

## Publisher’s Note

All claims expressed in this article are solely those of the authors and do not necessarily represent those of their affiliated organizations, or those of the publisher, the editors and the reviewers. Any product that may be evaluated in this article, or claim that may be made by its manufacturer, is not guaranteed or endorsed by the publisher.

## Supplementary Material

Supplementary Figure 1 Comparison of homozygote proportions for each *HLA-A* (red), *-B* (blue), and *-DRB1* (grey) allele in patients and controls.

Supplementary Figure 2 Comparison of frequencies of *HLA-A*03*, *A*32*, *B*27*, *B*39*, *B*55* and *DRB1*16* alleles showing significant differences among control group (black) and patient subgroups (mild/moderate: yellow; severe: red; critical: purple).

Supplementary Figure 3 Ranking of *HLA-A* (red), *-B* (blue) and *-C* (yellow) alleles observed in Iranian case-control cohort according to %Rank-based weak binders, %Rank-based strong binders, IC50-based weak binders and IC50-based strong binders, respectively.

Supplementary Figure 4 Density distributions of the numbers of SARS-CoV-2-derived peptides predicted by %Rank as strong binders to HLA-A (n_A_), HLA-B (n_B_), HLA-A and -B (n_AB_), and HLA-DR (n_DR_) molecules in patients (solid curves) and controls (dashed curves) of all ages and in those under 60 YO, respectively.

Supplementary Figure 5 Density distributions of the numbers of SARS-CoV-2-derived peptides predicted by IC50 (nm) as weak binders to HLA-A (n_A_), HLA-B (n_B_), HLA-A and -B (n_AB_), and HLA-DR (n_DR_) molecules in patients (solid curves) and controls (dashed curves) of all ages and in those under 60 YO, respectively.

Supplementary Figure 6 Density distributions of the numbers of SARS-CoV-2-derived peptides predicted by IC50 (nm) as strong binders to HLA-A (n_A_), HLA-B (n_B_), HLA-A and -B (n_AB_), and HLA-DR (n_DR_) molecules in patients (solid curves) and controls (dashed curves) of all ages and in those under 60 YO, respectively.

Supplementary Figure 7 Density distributions of the numbers of SARS-CoV-2-derived peptides predicted by %Rank as strong binders to HLA-A (n_A_), HLA-B (n_B_), HLA-A and -B (n_AB_), HLA-C (n_C_), HLA-A, -B and -C (n_ABC_), and HLA-DR (n_DR_) molecules **(A)** in three subgroups of patients (Mild/Moderate: solid curves in yellow; Severe: solid curves in red; Critical: solid curves in purple) and controls (dashed curves in black) and **(B)** in two subgroups of patients (Mild/Moderate: solid curves in yellow; Severe/Critical: solid curves in rose) and controls (solid curves in black) of all ages and in those under 60 YO, respectively. Note that n_C_ and n_ABC_ data were not available for controls.

Supplementary Figure 8 Density distributions of the numbers of SARS-CoV-2-derived peptides predicted by IC50 as strong binders to HLA-A (n_A_), HLA-B (n_B_), HLA-A and -B (n_AB_), HLA-C (n_C_), HLA-A, -B and -C (n_ABC_), and HLA-DR (n_DR_) molecules **(A)** in three subgroups of patients (Mild/Moderate: solid curves in yellow; Severe: solid curves in red; Critical: solid curves in purple) and controls (dashed curves in black) and **(B)** in two subgroups of patients (Mild/Moderate: solid curves in yellow; Severe/Critical: solid curves in rose) and controls (solid curves in black) of all ages and in those under 60 YO, respectively. Note that n_C_ and n_ABC_ data were not available for controls.

Supplementary Figure 9 Density distributions of the numbers of SARS-CoV-2-derived peptides predicted by IC50 as weak binders to HLA-A (n_A_), HLA-B (n_B_), HLA-A and -B (n_AB_), HLA-C (n_C_), HLA-A, -B and -C (n_ABC_), and HLA-DR (n_DR_) molecules **(A)** in three subgroups of patients (Mild/Moderate: solid curves in yellow; Severe: solid curves in red; Critical: solid curves in purple) and controls (dashed curves in black) and **(B)** in two subgroups of patients (Mild/Moderate: solid curves in yellow; Severe/Critical: solid curves in rose) and controls (dashed curves in black) of all ages and in those under 60 YO, respectively. Note that n_C_ and n_ABC_ data were not available for controls.
