# Supplementary figures and images for: Susceptibility and Severity of COVID-19 Are Both Associated With Lower Overall Viral–Peptide Binding Repertoire of HLA Class I Molecules, Especially in Younger People
Source: Front Immunol. 2022 Jul 7;13:891816. doi: 10.3389/fimmu.2022.891816 (PMC9331187; doi:10.3389/fimmu.2022.891816)

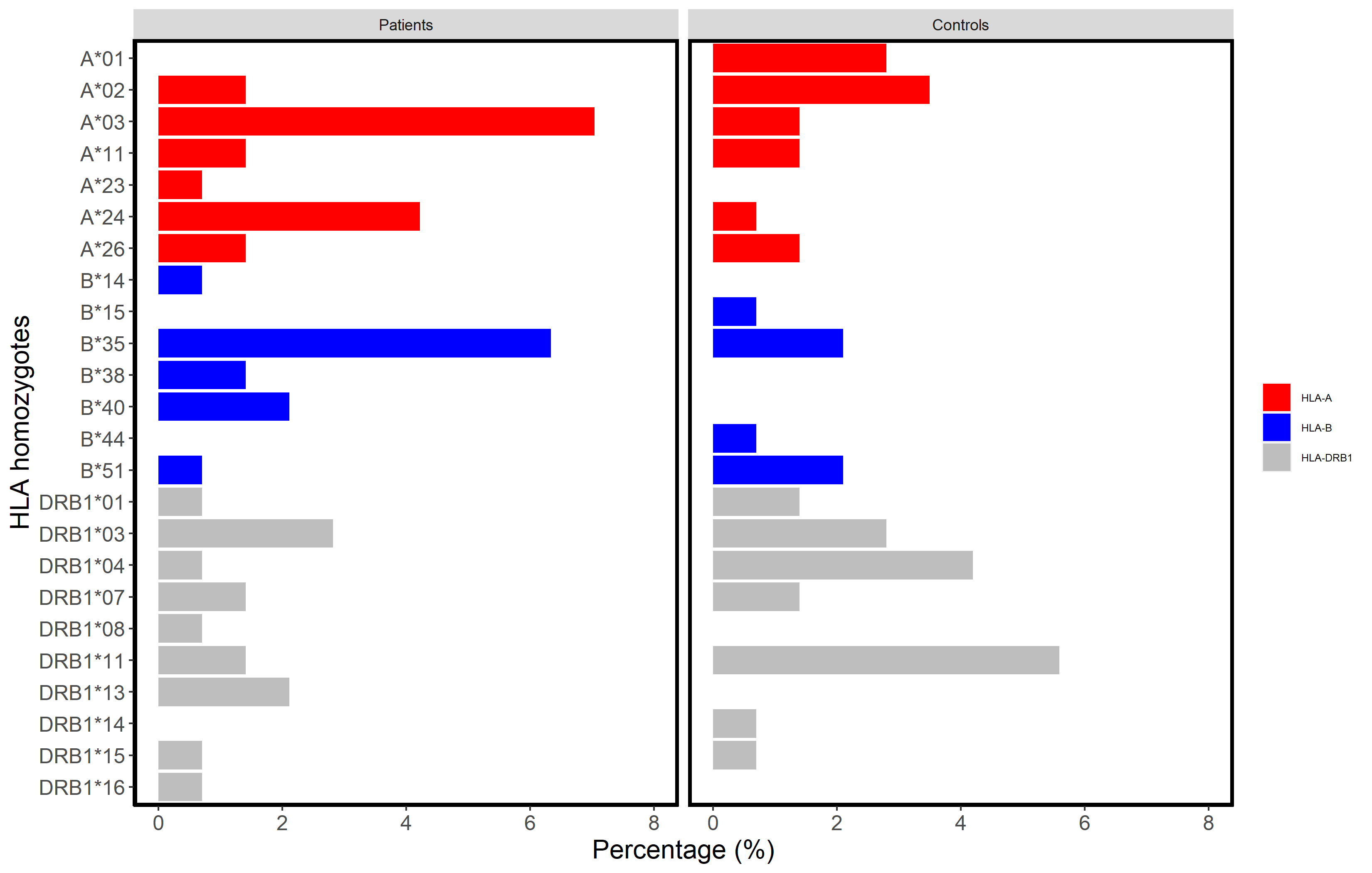

Supplement: Supplementary Figure 1 — Comparison of homozygote proportions for each HLA-A (red), -B (blue), and -DRB1 (grey) allele in patients and controls. [file Image_1.tif]

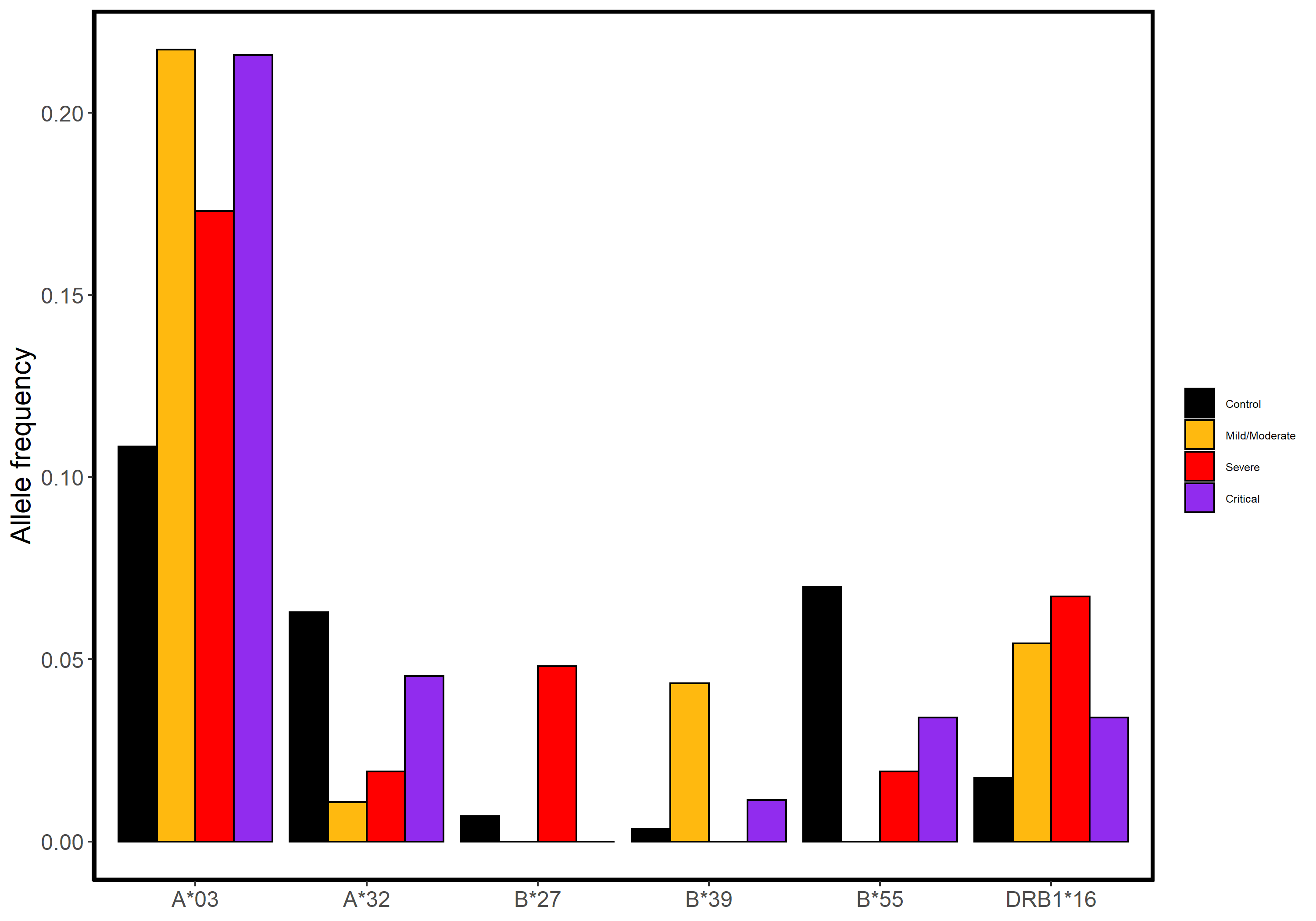

Supplement: Supplementary Figure 2 — Comparison of frequencies of HLA-A*03, A*32, B*27, B*39, B*55 and DRB1*16 alleles showing significant differences among control group (black) and patient subgroups (mild/moderate: yellow; severe: red; critical: purple). [file Image_2.tif]

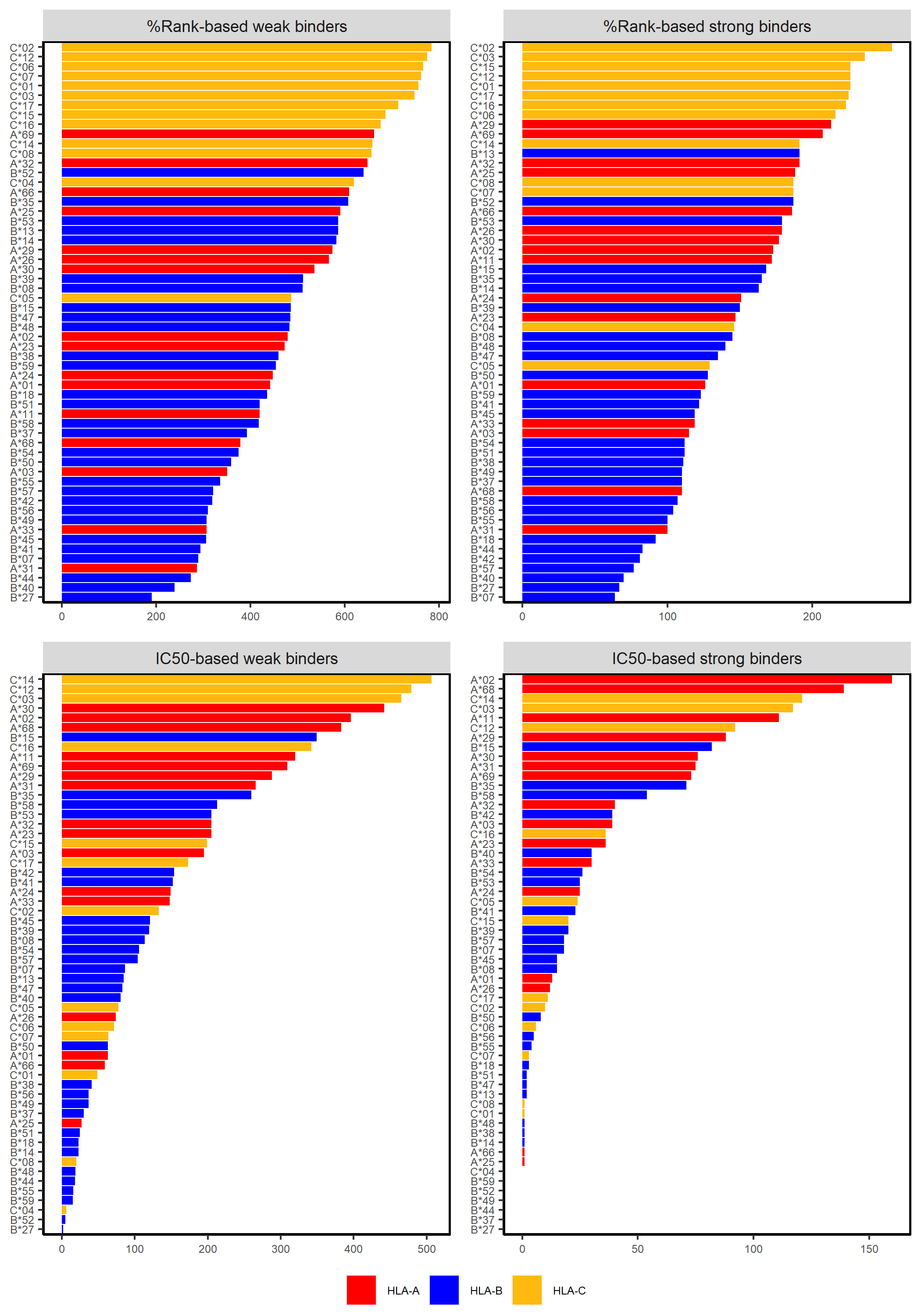

Supplement: Supplementary Figure 3 — Ranking of HLA-A (red), -B (blue) and -C (yellow) alleles observed in Iranian case-control cohort according to %Rank-based weak binders, %Rank-based strong binders, IC50-based weak binders and IC50-based strong binders, respectively. [file Image_3.tif]

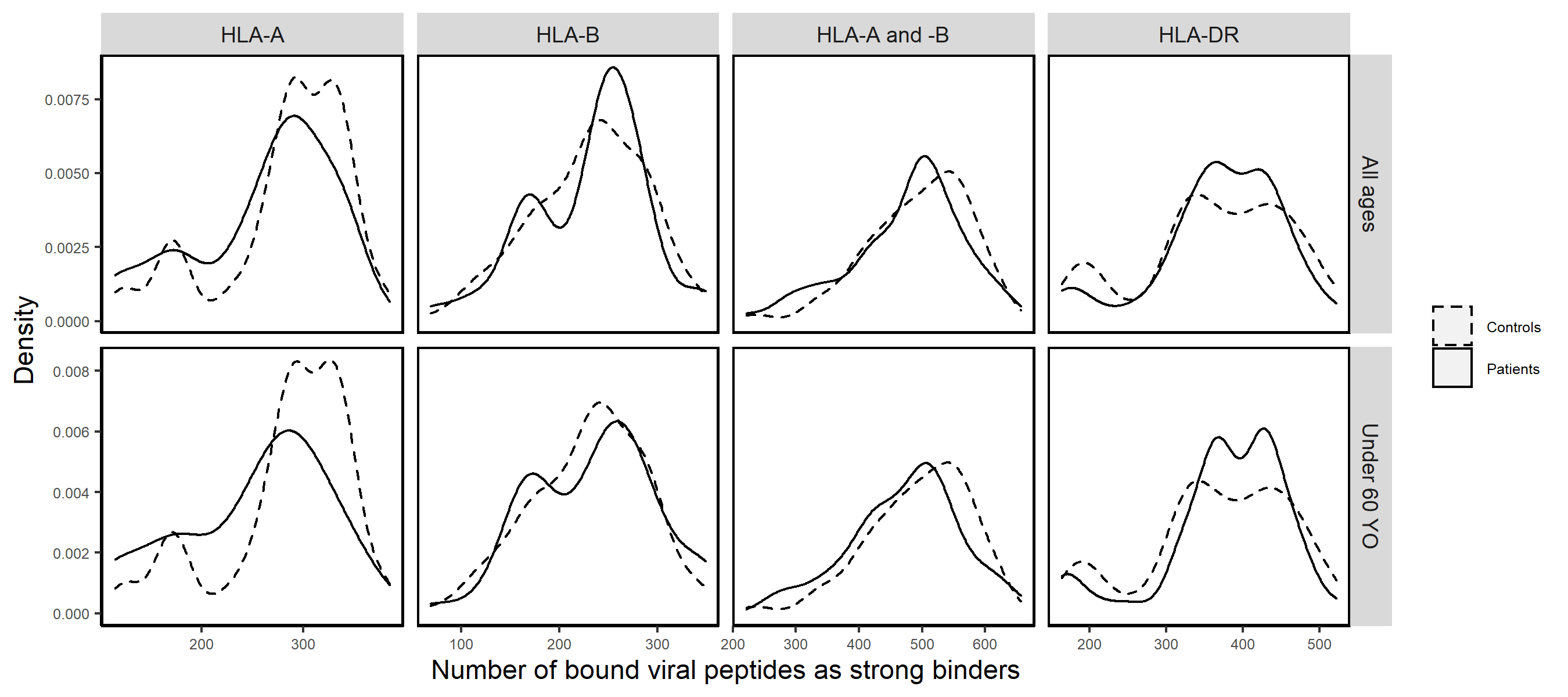

Supplement: Supplementary Figure 4 — Density distributions of the numbers of SARS-CoV-2-derived peptides predicted by %Rank as strong binders to HLA-A (nA), HLA-B (nB), HLA-A and -B (nAB), and HLA-DR (nDR) molecules in patients (solid curves) and controls (dashed curves) of all ages and in those under 60 YO, respectively. [file Image_4.tif]

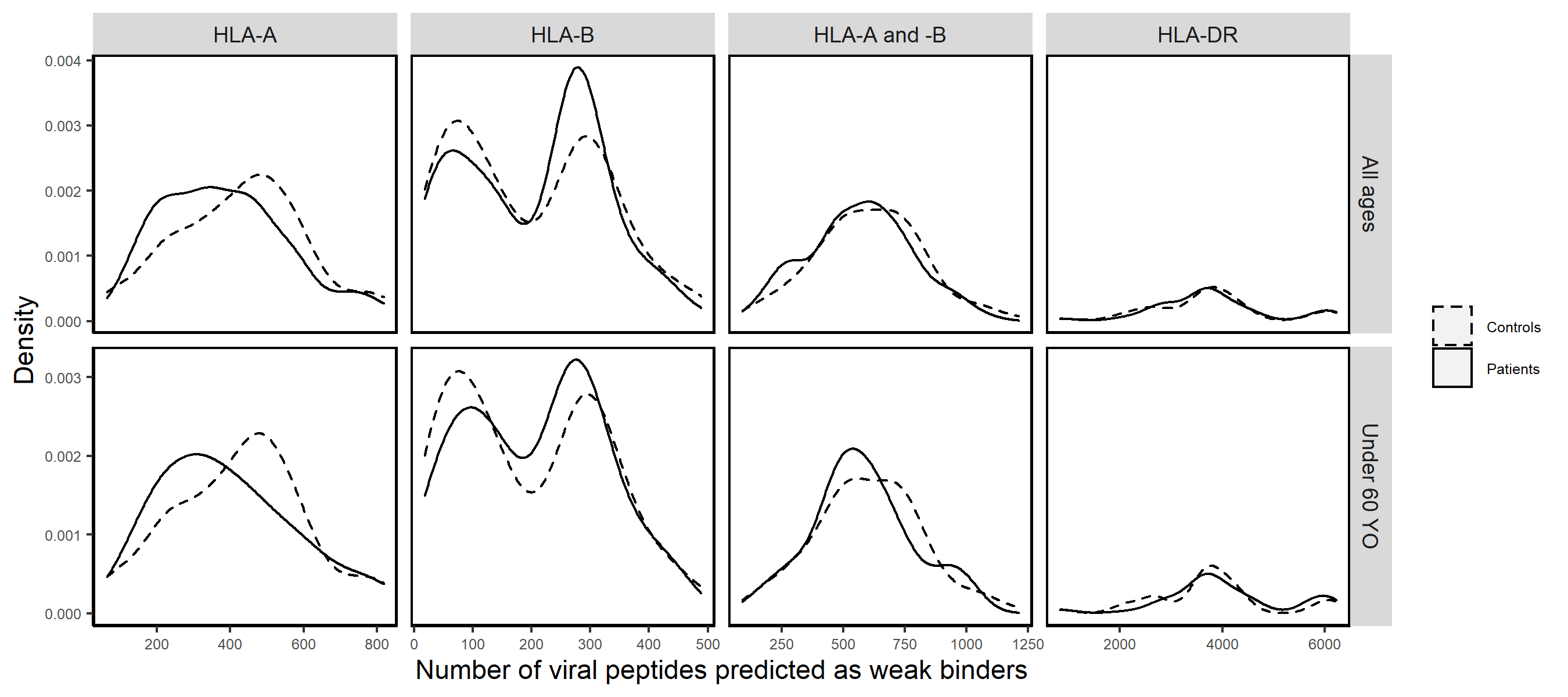

Supplement: Supplementary Figure 5 — Density distributions of the numbers of SARS-CoV-2-derived peptides predicted by IC50 (nm) as weak binders to HLA-A (nA), HLA-B (nB), HLA-A and -B (nAB), and HLA-DR (nDR) molecules in patients (solid curves) and controls (dashed curves) of all ages and in those under 60 YO, respectively. [file Image_5.tif]

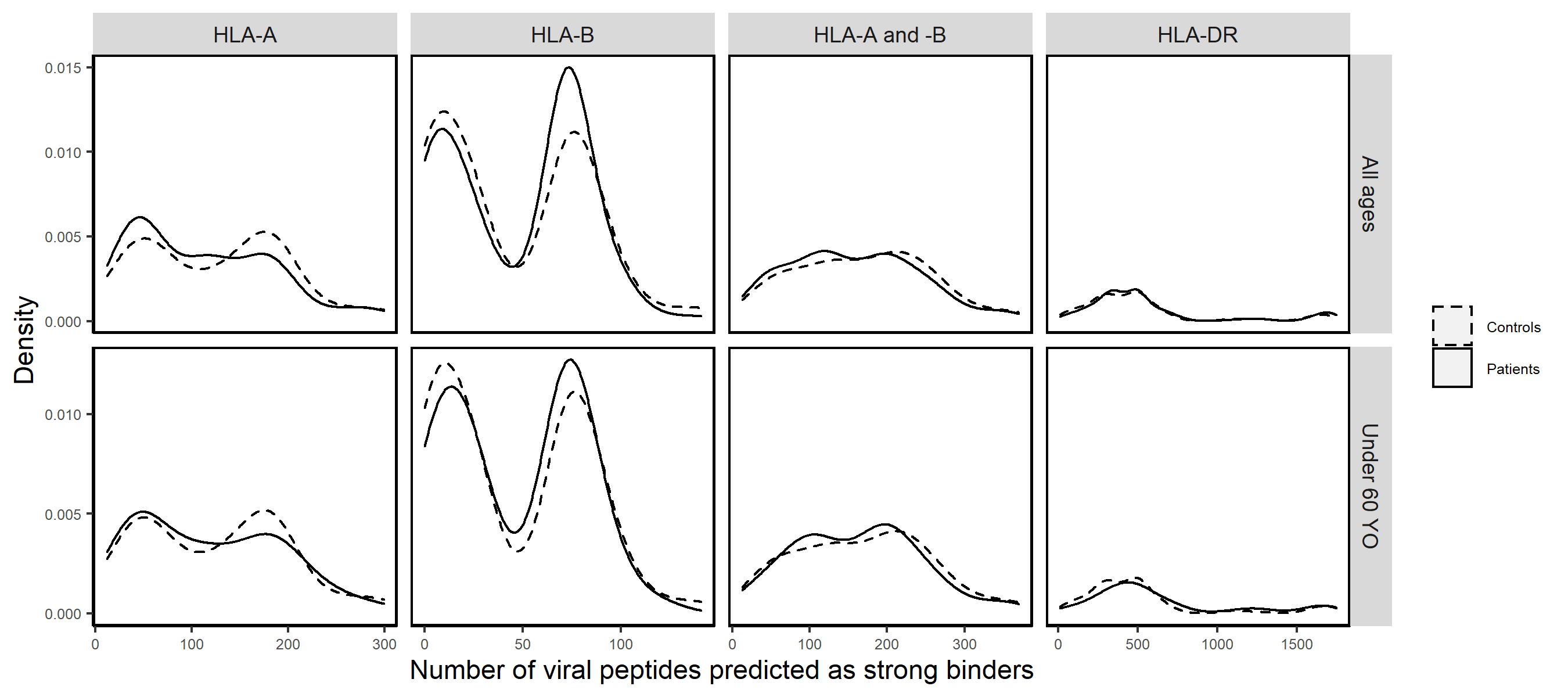

Supplement: Supplementary Figure 6 — Density distributions of the numbers of SARS-CoV-2-derived peptides predicted by IC50 (nm) as strong binders to HLA-A (nA), HLA-B (nB), HLA-A and -B (nAB), and HLA-DR (nDR) molecules in patients (solid curves) and controls (dashed curves) of all ages and in those under 60 YO, respectively. [file Image_6.tif]

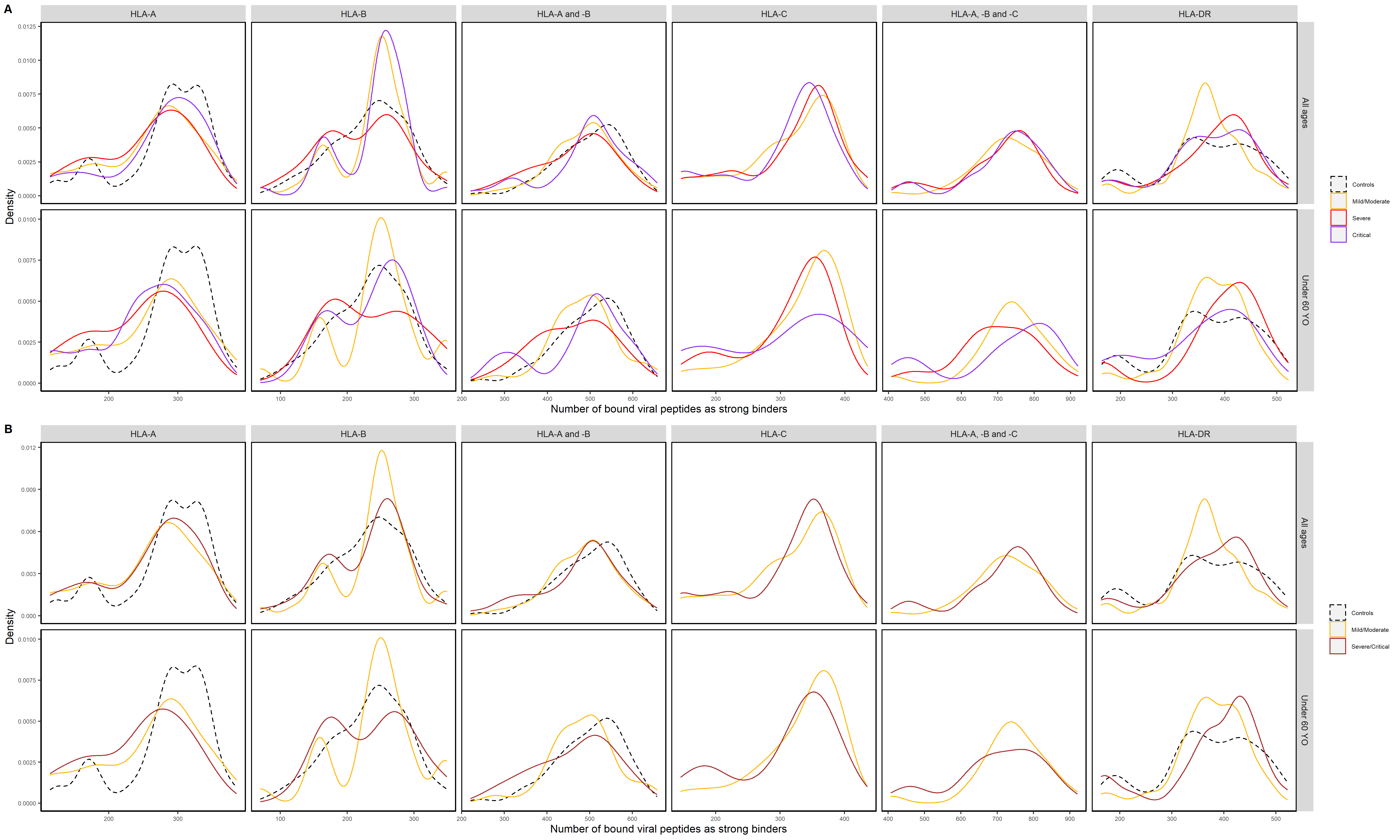

Supplement: Supplementary Figure 7 — Density distributions of the numbers of SARS-CoV-2-derived peptides predicted by %Rank as strong binders to HLA-A (nA), HLA-B (nB), HLA-A and -B (nAB), HLA-C (nC), HLA-A, -B and -C (nABC), and HLA-DR (nDR) molecules (A) in three subgroups of patients (Mild/Moderate: solid curves in yellow; Severe: solid curves in red; Critical: solid curves in purple) and controls (dashed curves in black) and (B) in two subgroups of patients (Mild/Moderate: solid curves in yellow; Severe/Critical: solid curves in rose) and controls (solid curves in black) of all ages and in those under 60 YO, respectively. Note that nC and nABC data were not available for controls. [file Image_7.tif]

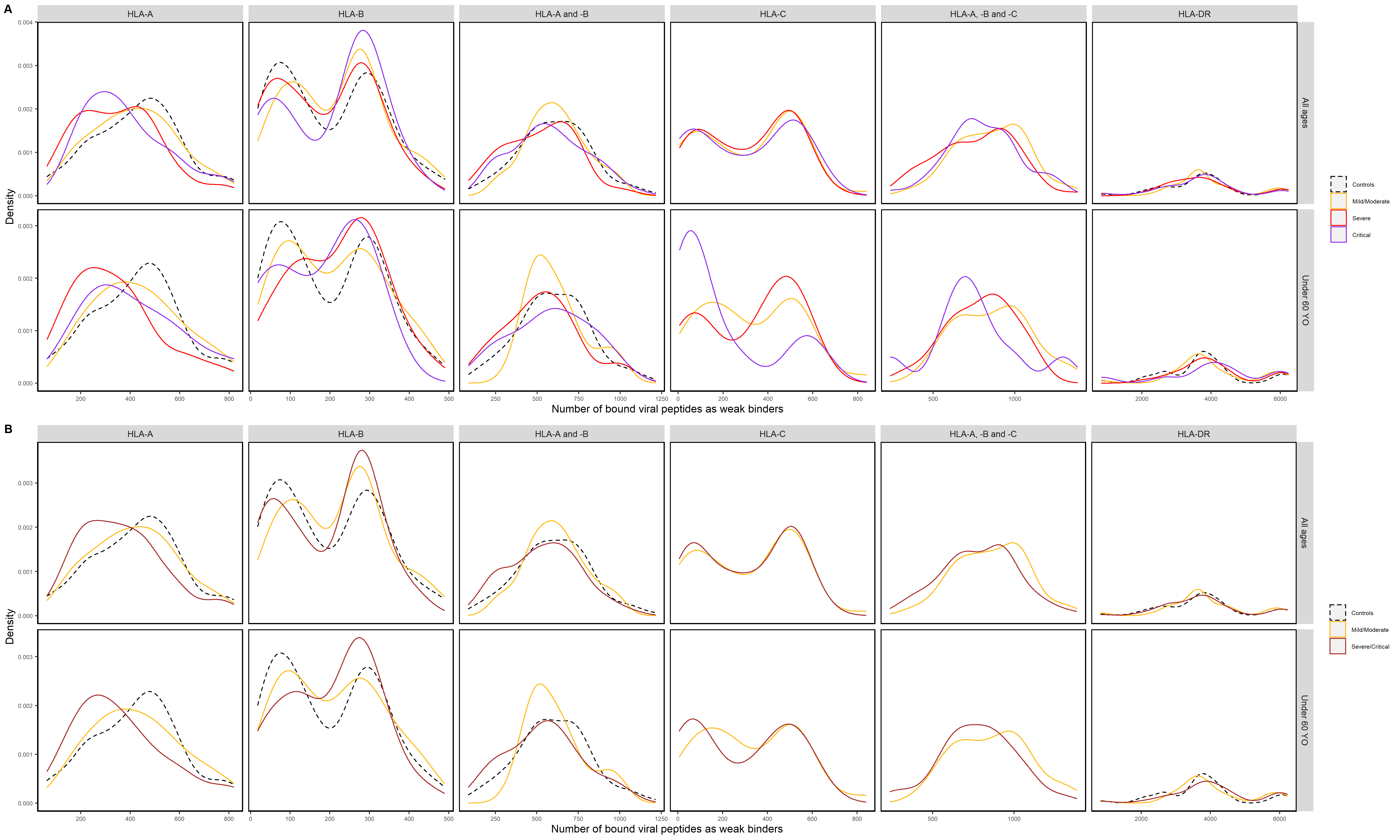

Supplement: Supplementary Figure 8 — Density distributions of the numbers of SARS-CoV-2-derived peptides predicted by IC50 as strong binders to HLA-A (nA), HLA-B (nB), HLA-A and -B (nAB), HLA-C (nC), HLA-A, -B and -C (nABC), and HLA-DR (nDR) molecules (A) in three subgroups of patients (Mild/Moderate: solid curves in yellow; Severe: solid curves in red; Critical: solid curves in purple) and controls (dashed curves in black) and (B) in two subgroups of patients (Mild/Moderate: solid curves in yellow; Severe/Critical: solid curves in rose) and controls (solid curves in black) of all ages and in those under 60 YO, respectively. Note that nC and nABC data were not available for controls. [file Image_8.tif]

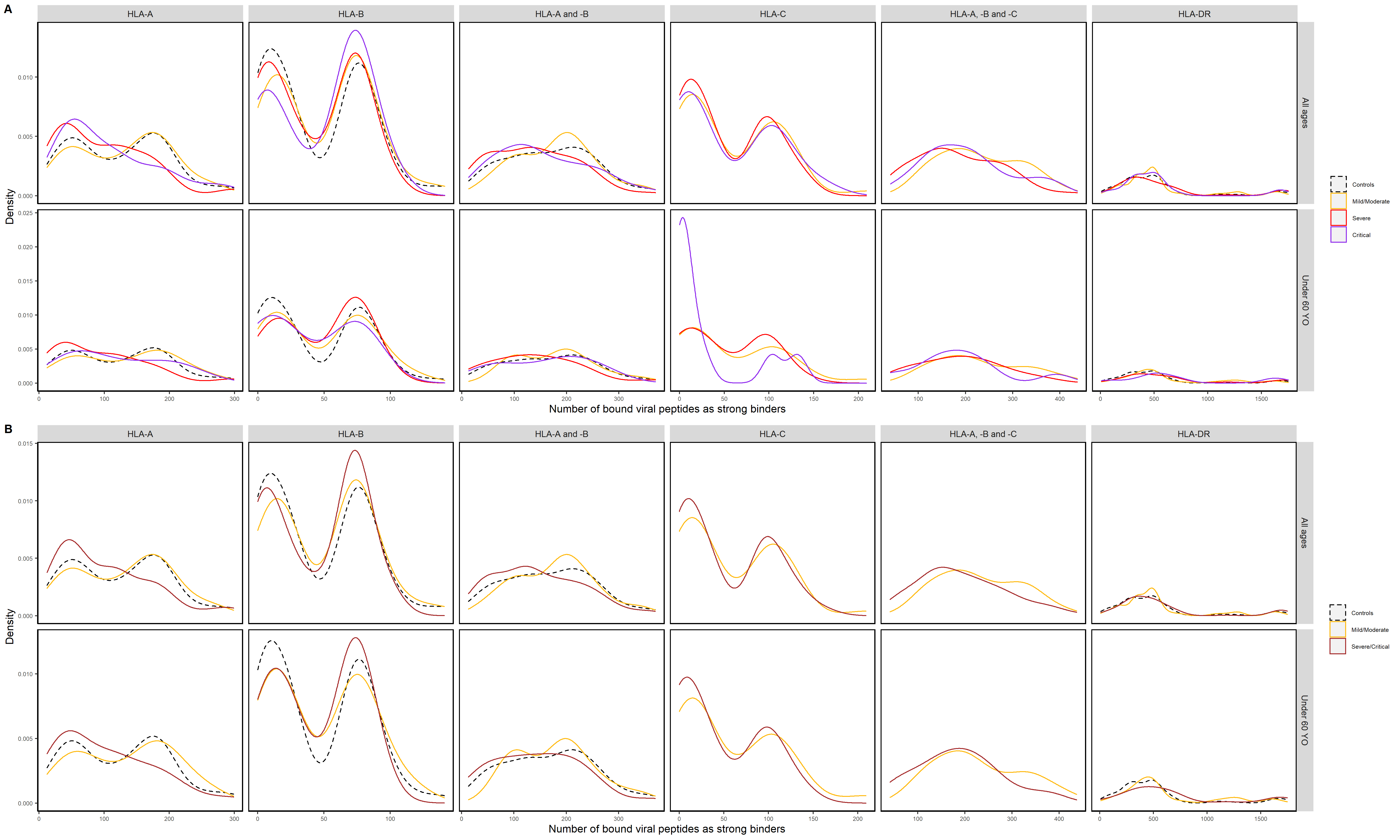

Supplement: Supplementary Figure 9 — Density distributions of the numbers of SARS-CoV-2-derived peptides predicted by IC50 as weak binders to HLA-A (nA), HLA-B (nB), HLA-A and -B (nAB), HLA-C (nC), HLA-A, -B and -C (nABC), and HLA-DR (nDR) molecules (A) in three subgroups of patients (Mild/Moderate: solid curves in yellow; Severe: solid curves in red; Critical: solid curves in purple) and controls (dashed curves in black) and (B) in two subgroups of patients (Mild/Moderate: solid curves in yellow; Severe/Critical: solid curves in rose) and controls (dashed curves in black) of all ages and in those under 60 YO, respectively. Note that nC and nABC data were not available for controls. [file Image_9.tif]
